# The effectiveness and safety of plum-blossom needle therapy for Tourette syndrome: study protocol for a randomized controlled trial

**DOI:** 10.1186/s13063-015-0873-0

**Published:** 2015-07-29

**Authors:** Jinna Yu, Yongming Ye, Shanshan Li, Jun Liu, Yanbing Zhai, Min Zhang, Zhishun Liu

**Affiliations:** Acupuncture Department, Guang’anmen Hospital, China Academy of Chinese Medical Sciences, No. 5, Beixiange Street, 100053 Xicheng District Beijing, China

**Keywords:** Acupuncture, Plum-blossom needle, Tourette syndrome, Randomized controlled trial

## Abstract

**Background:**

Previous studies have indicated that acupuncture can alleviate the symptoms of Tourette syndrome (TS), but the evidence is insufficient. So far, there have been no reports on plum-blossom needle therapy for TS. Here we present a protocol for a randomized controlled trial using plum-blossom needle therapy to treat TS.

**Methods/design:**

Sixty patients will be randomly allocated into either the plum-blossom needle therapy group or the habit reversal training (HRT) group. All patients in each group will be given 12 weeks of treatment, with follow-up at the 24th week. The primary outcome measure will be the mean change from baseline in the total tic score on the Yale Global Tic Severity Scale (YGTSS) at the 12th week. Secondary outcome measures will include the scores on the TS Clinical Global Impression Scale (CGI) and the mean changes from baseline in the YGTSS score and the Children and Adolescents’ Quality of Life Scale (CAQOL) at other time points. Safety will also be evaluated.

**Discussion:**

This trial will evaluate the effectiveness and safety of plum-blossom needle therapy for TS compared with HRT. A limitation of this trial is that patients and acupuncturists cannot be blinded.

**Trial registration:**

ClinicalTrials.gov Identifier: NCT02403258 (Date of registration: March 31, 2015).

## Background

Tourette syndrome (TS) is a childhood-onset chronic neuropsychiatric disorder characterized by multiple motor tics and one or more vocal tics, with a duration of at least 1 year [1]. Population-based studies indicate that TS affects approximately 0.3–1 % of children in Western countries [2–6] and 0.43–0.56 % of children in China [7, 8]. TS is two to four times more common in boys than girls [5]. The majority of TS cases have comorbid conditions such as attention-deficit hyperactivity disorder, obsessive-compulsive disorder, mood disorders, learning disorders, self-injurious behavior, and other behavior disorders. TS and its complications seriously influence the quality of patients’ lives [9, 10].

The guidelines developed by the European Society for the Study of Tourette syndrome (ESSTS) in 2011 recommended that TS could be treated by behavioral therapy (habit reversal training or exposure with response prevention) and medications including alpha-adrenergic agents, typical antipsychotics, atypical antipsychotics, and benzamides [11, 12]. Although there is strong evidence in favor of pharmacological intervention, it is also known that medications can rarely eradicate tics completely and are always associated with unwanted side effects. The most commonly observed adverse events of typical antipsychotics are extrapyramidal reactions, neuroleptic malignant syndrome, drowsiness, restlessness, and sexual dysfunction; sulpiride may lead to sustained sedation or drowsiness [13–15].

Acupuncture is a conventional Traditional Chinese Medicine method that dates back thousands of years, and it has specifically been used to treat TS for many years. A Chinese systematic review of six randomized controlled trials published in 2010 revealed that acupuncture was probably more effective for TS than medications [16]. However, the included studies were all published in Chinese, with the limitations of small sample sizes and poor design quality; the systematic review could not provide sufficient evidence to support the effectiveness of acupuncture in treating TS.

At present, many parents in China seek treatment from Traditional Chinese Medicine practitioners for their children with TS. However, maintaining a fixed position during conventional acupuncture is difficult for children, and the pain associated with conventional acupuncture leads to poor compliance. Plum-blossom needle therapy is a method of shallow insertion of multiple needles into the skin. The treatment instrument is made of five or seven stainless-steel needles arranged in a plum-blossom-shaped pattern, hence the name ‘plum-blossom needle.’ Plum-blossom needle therapy treats diseases by tapping specific skin areas or acupoints corresponding to the treatment of different illnesses based on the meridian theory. Our pilot study in which we used self-made blunt-pointed plum-blossom needles showed that plum-blossom needle therapy can relieve the symptoms of TS. The treatment was well accepted by children with TS because of its mild pain as the needles did not pierce the skin.

This protocol is designed for phase II of our trial. The objective of this trial is to evaluate the effectiveness and safety of plum-blossom needle therapy for treating TS.

## Methods/design

### Study design

This protocol is for a prospective randomized controlled trial. Randomization will be performed by the Good Clinical Practice (GCP) office of Guang’anmen Hospital of the China Academy of Chinese Medical Sciences. Statistics Analysis System software version 9.1.3 (SAS Institute, Cary, NC, USA) will be used to generate the random allocation sequence with a block of four. Opaque sealed envelopes will be used. Patients' sequence numbers are printed on the envelope surface. The random allocation sequence numbers and group names are inside the envelope. The randomization program will be saved by an appointed staff member in the GCP office who will not take part in this trial.

**Fig. 1:**
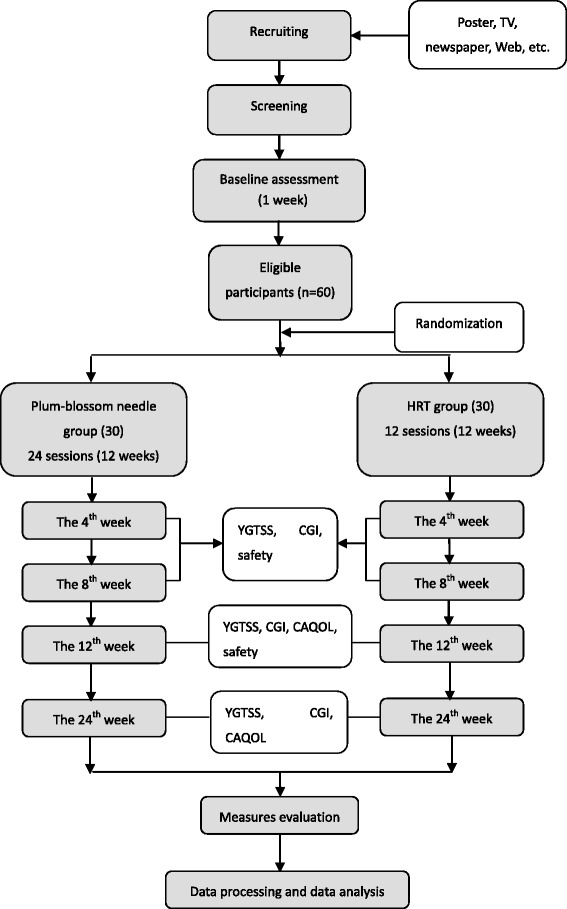
Flowchart of the proposed trial procedure

The trial will be conducted in Guang’anmen Hospital over 3 years from 1 January 2015 to 30 May 2018. We plan to recruit participants via advertisements in newspapers, television, websites, and posters. Eligible participants diagnosed with TS by a psychiatrist will be randomly assigned into the plum-blossom needle therapy group or the HRT group at a 1:1 ratio. A flowchart of the trial procedure is presented in Fig. 1.

**Fig. 2:**
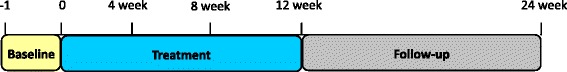
Proposed timeline of the trial

The duration of the study will be 25 weeks, including a baseline period of 1 week (week 0–1), a treatment period of 12 weeks (week 1–12), and a follow-up period of 12 weeks (week 13–24). The timeline of the trial is presented in Fig. 2.

### Ethical approval

All procedures will be explained to patients and their guardians; the informed consent form will be reviewed and signed by each participant and their guardian before randomization. The trial protocol is in accordance with the principles of the Declaration of Helsinki (version Edinburgh 2000) and has been approved by the Ethics Committee of Guang’anmen Hospital, China Academy of Chinese Medical Sciences (ethics approval no. 2014EC104).

### Participants

A target sample of s60 participants with TS will be recruited in the trial.

#### Inclusion criteria

1. Participants must meet the diagnostic criteria of the Diagnostic and Statistical Manual of Mental Disorders of the American Psychiatric Association (DSM-IV-TR) for TS: Both multiple motor and one or more vocal tics have been present at some time during the illness, although not necessarily concurrently. The tics occur many times (usually in bouts) nearly every day or intermittently throughout a period of more than 1 year; during this period there was never a tic-free period of more than 3 consecutive months. Onset was before the age of 18 years. The disturbance is not due to the direct physiological effects of a substance (e.g., stimulants) or a general medical condition (e.g., Huntington’s chorea or postviral encephalitis) [1].
2. Age 7 to 18 years.
3. Participants must agree to participate in the trial, and both guardians and subjects must sign written informed consent.

#### Exclusion criteria

1. TS patients with concurrent severe problems involving the heart, liver, or kidney, or with hyperthyroidism.
2. After evaluation by psychiatrists based on the Kiddie-Sads-Present and Lifetime Version, those who have comorbid conditions such as mental retardation, pervasive developmental disorder, schizophrenia, manic episode, bipolar disorder, anxiety and depression, or a specific learning disorder will be excluded.
3. Tic symptoms caused by drugs.
4. Subjects who are using pharmacological or non-pharmacological treatment for TS have to be free from that for at least 2 weeks prior to baseline assessment.
5. Patients with a history of psychotropic substance or alcohol use during the 3 months preceding screening.
6. Patients with a history of non-responsiveness to HRT and those participating in another clinical trial.

### Intervention

Lifestyle education will be taught to all parents/guardians of patients, including the stability and unity of the family, not being strict with their children, and reducing patients’ psychological burden. Patients will also be required to abstain from exciting novels and films. All participants must not receive any other therapy for TS during the trial.

### Plum-blossom needle therapy group

**Fig. 3:**
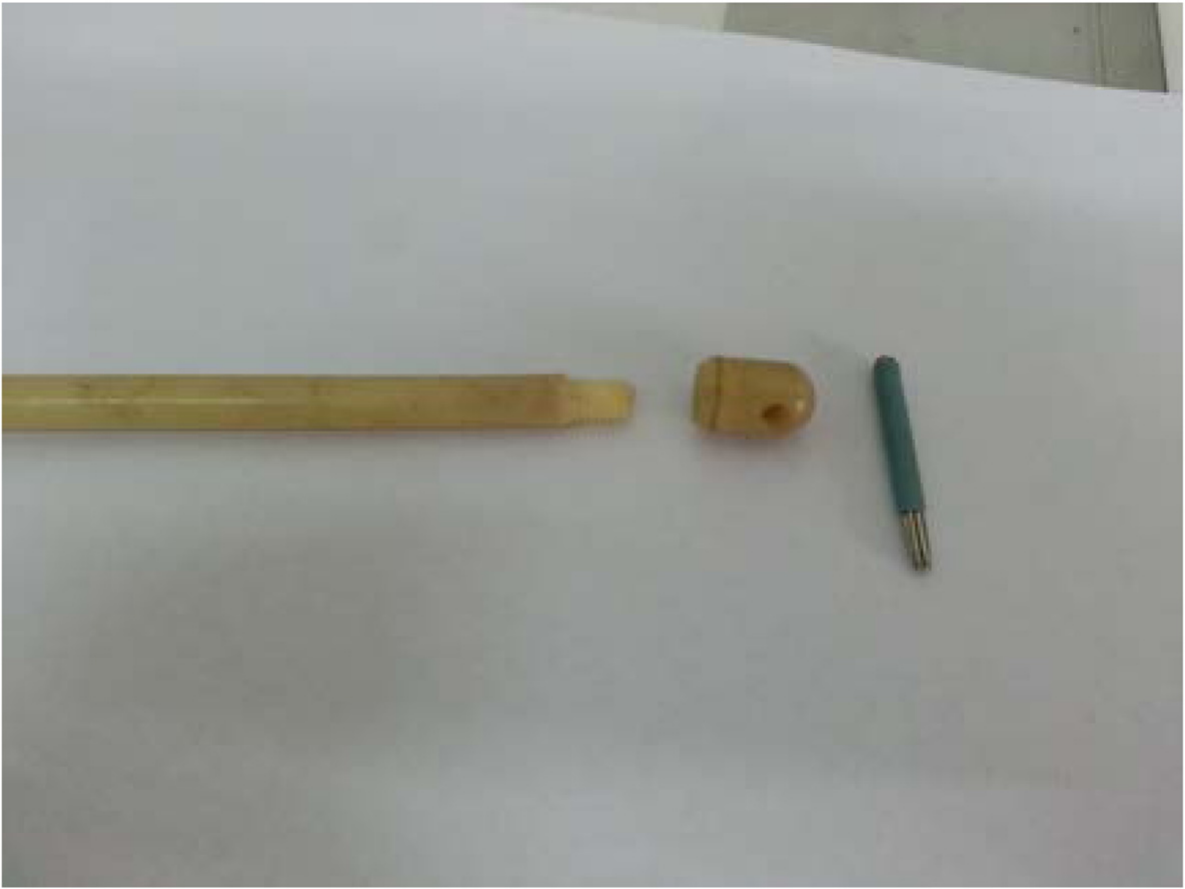
Composition of the plum-blossom needle with a detachable head. Each needle head will be used for a specific patient and will be sterilized at high pressure after each use

**Fig. 4:**
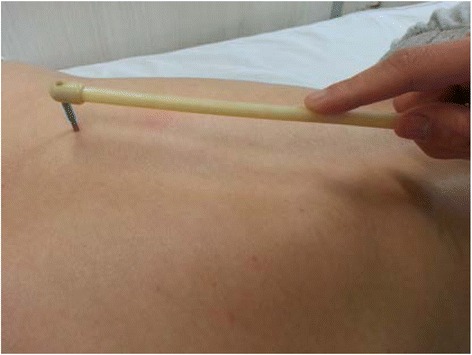
Manipulation of the plum-blossom needle

The acupoints were selected based on our pilot study and consensus with experienced acupuncture experts. The points will be DU20 (Baihui), DU16 (Fengfu), GB20 (Fengchi), EX-HN5 (Taiyang), BL15 (Xinshu), BL18 (Ganshu), and BL23 (Shenshu). Self-made head-detachable sterile plum-blossom needles will be used in this trial (see Fig. 3). After the skin has been wiped with 75 % alcohol, plum-blossom needles will be tapped perpendicularly on the skin at the selected acupoints with wrist force and then lifted immediately (see Fig. 4). Each point will be tapped repeatedly for 30 s and totally 6 min for each session. Acupuncturists should tap the points gently to make patients feel only a little pain and stop when the skin gets flushed but is not bleeding. The treatment will be two sessions per week for 12 weeks (24 sessions in all). Treatments will be done by acupuncturists with over 2 years of working experience and academic credentials above an undergraduate degree; they will be trained specifically for this protocol prior to the trial.

### HRT group

HRT will consist of the following: (1) self-monitoring, (2) competing responses, (3) relaxation training, and (4) contingency management. Self-monitoring will be done to increase patients’ awareness of premonitory sensations or urges and to obtain detailed descriptions of varied tic symptoms. Patients will be provided with charts in which they can record the occurrence of tics, preceding sensations/urges, and the muscles and movements involved in a tic. Competing response training will involve each patient being trained to perform more appropriate physical responses when they feel the urge to perform tics, thereby preventing tic occurrence. For example, patients with arm jerking tics will be trained to contract the tic-opposing muscles when they are aware of the early sensations or urges preceding tics. For vocal tics, competing responses such as closing the mouth and breathing slowly through the nose will be used to avoid occurrence of tics. For complex tics, competing responses for individual components will be developed first and then chained. Patients will also be taught the relaxation techniques of thoracic breathing and diaphragmatic breathing. Treatment will typically be started with the patients’ most bothersome tics, unless the patient and clinician determine that competing responses for a less bothersome tic would be easier to implement before moving on to a more bothersome/severe/complex tic [17, 18]. The HRT training will be given weekly for 12 weeks (12 sessions in total) by a special rehabilitation therapist. The first two sessions will be 1.5 h, and the remaining sessions will be 1 h for each treatment.

### Outcome measures

The primary outcome measure will be the mean change from baseline in the total tic score on the Yale Global Tic Severity Scale (YGTSS) at the 12th week.

Secondary outcome measures will include the mean change from baseline in the YGTSS score at the 4th, 8th, and 24th week, the mean score on the TS Clinical Global Impression Scale (CGI) at the 4th, 8th, 12th, and 24th week, and the mean score change compared with baseline on the Children and Adolescents’ Quality of Life Scale (CAQOL) at the 12th and 24th week.

Adverse events including fainting, stabbing pain during acupuncture, fatigue, insomnia, appetite change, worsening of symptoms, local infections, and other side effects related to acupuncture will be recorded in detail by patients, their parent/guardian, acupuncturists, and interviewers.

The outcome assessments will be done by research assistants who are blinded to the clinical management.

### Quality control and data collection

All staff involved in the trial will receive training before the trial commences. The training program will include case screening, intervention method, evaluation of outcome measures, completion of case report forms (CRFs), and data management.

Two data managers with medical backgrounds will check the data recorded in the CRFs. After a data review, two research assistants will independently input the data using EpiData software (EpiData Association, Odense, Denmark). The data will then be submitted to a statistician independent from the research team for final analysis.

### Sample size and statistical analysis

The reduction in YGTSS score in the pilot trial after 12 weeks of treatment in the plum-blossom needle therapy group and HRT group was 13.12 ± 5.40 (mean ± standard deviation) and 9.50 ± 3.20 (mean ± standard deviation), respectively. Using a one-sided superiority test (α = 0.025), 30 patients will be required in each group to give a power of 80 %, allowing for a 20 % dropout (1:1 ratio).

The outcome parameters of randomized patients will be analyzed on an intention-to-treat basis. Missing data will be replaced by the last entry. All outcome measures will be analyzed by two-sided tests. All significance levels will be established at 0.05. Continuous data will be represented by the mean, standard deviation, median, minimum value, and maximum value; categorical data will be represented by percentages. A *t*-test or nonparametric test will be used for continuous data. A chi-square test or Fisher’s exact test will be used for categorical data. For analysis of safety, the chi-square test or Fisher’s exact test will be done to compare the incidence of side effects between the two groups. Analyses will be performed using SPSS version 13.0 software (SPSS Inc., Chicago, IL, USA).

## Discussion

Traditional plum-blossom needles pierce the skin when treating disease. However, to improve patient compliance in this proposed trial, we will use self-made blunt-pointed plum-blossom needles that will not pierce the skin and hence will alleviate the pain during treatment.

The primary aim of this study is to evaluate the effectiveness of plum-blossom needle therapy for TS. As the blunt-pointed plum-blossom needles will not pierce the skin, we cannot feasibly conduct sham plum-blossom needle therapy or placebo as a control group. HRT, recommended as an effective and safe TS treatment by the ESSTS, is the most extensively researched behavioral treatment for tics. Hence, comparing plum-blossom needle therapy with HRT will reflect the effectiveness and clinical value of plum-blossom needle therapy for TS. Most studies of HRT for TS have 10–14-week-long treatment, with follow-up data at 1 month to 1 year, mostly 3 months post-treatment. Thus, it is appropriate to conduct the follow-up visit at the 24th week after the last-time treatment [12, 19–21]. We will therefore measure the primary outcome at the 12th week, but we will also observe the changes in outcome measurements at the 4th, 8th, and 24th week to observe the time of onset and maintenance of the effect produced by plum-blossom needle therapy. In this trial, patients and acupuncturists will not be blinded, which means bias cannot be avoided. However, we can partially avoid biases caused by expectations and subjective assessment via rigid randomization, concealment of the allocation, and blinding of outcome assessors and statisticians. Otherwise, the outcome of this trial could not exclude the possible placebo effect of plum-blossom needle therapy, so we would not know its true efficacy.

## Trial status

The first participant was included on 3 January 2015. To date, two participants have been recruited.

### Consent

Written informed consent will be obtained from the patient(s) for publication of this manuscript and accompanying images. A copy of the written consent is available for review from the Editor-in-Chief of this journal.

## Abbreviations

TS: Tourette syndrome
HRT: Habit reversal training
YGTSS: Yale Global Tic Severity Scale
CGI: Clinical Global Impression Scale
CAQOL: Children and Adolescents’ Quality of Life Scale
ESSTS: European Society for the Study of Tourette Syndrome
DSM: Diagnostic and Statistical Manual of Mental Disorders
